# Deintensification Strategies Using Proton Beam Therapy for HPV-Related Oropharyngeal Cancer

**DOI:** 10.14338/IJPT-20-00073.1

**Published:** 2021-06-25

**Authors:** Mauricio E. Gamez, Daniel J. Ma

**Affiliations:** 1 Department of Radiation Oncology, The Ohio State University, Columbus, OH, USA; 2 Department of Radiation Oncology, Mayo Clinic, Rochester, MN, USA

**Keywords:** HPV, oropharyngeal cancer, deintensification, protons

## Abstract

Oropharyngeal cancers related to the human papillomavirus are a growing segment of head and neck cancers throughout the world. These cancers are biologically and demographically unique with patients presenting at younger ages and with more curable disease. This combination of factors heightens the importance of normal tissue sparing because patients will live a long time with treatment sequelae. Proton therapy has demonstrated benefits in reducing normal tissue exposure, which may lead to less toxicity, a higher quality of life, less immunologic suppression, and lower cost. Research investigating deintensified radiation volumes and doses are also underway. These deintensification studies synergize well with the beam characteristics of proton beam therapy and can decrease that already reduced normal tissue exposure enabled by proton therapy. Future studies should refine patient selection to best allow for volume and dose reduction paired with proton therapy.

## Introduction

As of 2020, oropharyngeal squamous cell carcinomas (OPSCCs) constitute most new head and neck cancer diagnoses within the United States. The annual number of OPSCC cases is projected to rise from a baseline of 20,214 cases in 2016 to an estimated 30,629 cases by 2029, a 51.5% increase in less than 2 decades [1]. Of those OPSCC cases, more than 70% of them will be related to the human papillomavirus (HPV-OPSCC) [2]. Given the decades-long carcinogenesis process and the slower acceptance of HPV vaccination, particularly among boys, that trend is expected to continue for the foreseeable future [3]. Therefore, HPV-OPSCC is an emerging cancer epidemic that will persist in the oncologic landscape for decades to come.

In addition, HPV-OSCC represents a demographically and biologically distinct disease compared with historic head and neck squamous cell carcinomas. Patients with HPV-OPSCC tend to be younger and have fewer medical comorbidities when compared with patient cohorts from other head and neck cancer subtypes [4]. Furthermore, in vitro and in vivo experiments demonstrate that these tumors are more sensitive to radiation and chemotherapy compared with historic HPV-negative head and neck squamous cell carcinomas. These factors lead to high survival rates among patients with HPV-OPSCC, particularly in nonsmokers. Because these patients tend to be young and otherwise healthy, they will live with their treatment sequela for a long time.

Radiation therapy serves as the backbone for most of the treatment options of HPV-OPSCC. Standard treatments, consisting of either chemoradiation or surgery followed by adjuvant treatment, offer high cure rates, with survival rates exceeding 90% for nonsmokers [5]. Nevertheless, nearly a third of patients will live with debilitating effects of treatment, including xerostomia, dysphagia, neuropathy, neck fibrosis, or osteoradionecrosis [5]. The rate of depression after standard treatments can reach 50% [6]. Improved treatment delivery strategies are thus required to reduce radiation-related toxicity for these patients while preserving historic disease control rates.

Many of the long-term effects associated with HPV-OPSCC arise from cumulative radiation dose to healthy tissue. Numerous research efforts aim to deintensify treatment by reducing radiation dose, decreasing radiation target volumes, combining novel treatment modalities, or combinations of these approaches. Multiple strategies exist for reducing toxicity from HPV-OPSCC treatment. The unique qualities of proton-beam therapy (PBT) for healthy tissue sparing allow for significant synergies with treatment deintensification efforts. This article explores the role of PBT in HPV-OPSCC, research efforts for treatment deintensification, and how proton therapy can aid current deintensification strategies. A summary of representative deintensification strategies can be found in the Table.

**Table.** Representative deintensification trials relevant for proton therapy.

**Strategy**	**Trial**	**Phase**	**Treatment**
Proton therapy	Randomized trial of IMPT versus IMRT for the treatment of oropharyngeal cancer of the head and neck (NCT01893307) [11]	Phase II/III	Randomized protons versus photons
	TORPEdO; A phase III trial of intensity-modulated proton beam therapy versus intensity-modulated radiotherapy for multi-toxicity reduction in oropharyngeal cancer (ISRCTN16424014) [46]	Phase III	Randomized protons versus photons
	Photon therapy versus proton therapy in early tonsil cancer (ARTSCAN V) (NCT03829033) [47]	Phase III	Randomized protons versus photons for early stage tonsil cancer
Volume deintensification	AVOID trial; A single-arm phase II study of post-transoral robotic surgery (TORS) alone to the primary tumor site and selective neck dissection (SND) followed by adjuvant radiation therapy (+/− chemotherapy) to the regional nodes for advanced stage, human papilloma virus (HPV) positive, oropharyngeal cancer (NCT02159703) [23, 24]	Single arm Phase II	Mucosal sparing to the primary tumor site after TORS
	A study of mucosal sparing proton beam therapy (PBT) in resected oropharyngeal tumors (NCT02736786) [21, 22]	Observational	Mucosal sparing to the primary tumor site after TORS
	Eliminating postoperative radiation to the pathologically node-negative neck [26]	Single-arm phase II	Eliminating adjuvant radiation therapy to a dissected, pathologically negative contralateral neck
	Nab-paclitaxel and carboplatin followed by response-based local therapy in treating patients with stage III or IV HPV-related oropharyngeal cancer (OPTIMA) (NCT02258659) [31, 32]	Phase II	Induction chemotherapy followed by response-stratified treatment; RT/CRT was limited to the first echelon of uninvolved nodes
Radiation dose reduction, definitive	De-intensified chemoradiotherapy for human papillomavirus–associated oropharyngeal squamous cell carcinoma [33]	Single-arm phase II	Definitive chemoradiation therapy to 60 Gy in 30 fractions
	NRG HN002; Reduced-dose intensity-modulated radiation therapy with or without cisplatin in treating patients with advanced oropharyngeal cancer (NCT02254278) [34, 35]	Randomized phase II	60 Gy chemoradiation versus 60 Gy 6 fractions/wk
Radiation dose reduction, postinduction	Induction chemotherapy followed by chemoradiotherapy for head and neck cancer; Paclitaxel and carboplatin before radiation therapy with paclitaxel in treating HPV-positive patients with stage III-IV oropharynx, hypopharynx, or larynx cancer (NCT01716195, NCT02048020) [50, 51]	Phase II	Carboplatin/paclitaxel followed by 54 Gy or 60 Gy for responders and non-responders, respectively, with weekly paclitaxel
	ECOG 1308; Induction chemotherapy followed by cetuximab and radiation in HPV-associated resectable stage III/IV oropharynx cancer (NCT01084083) [38, 39]	Phase II	Cisplatin, paclitaxel, and cetuximab followed by 54 Gy versus 69.3 Gy depending upon response
Radiation dose reduction, postsurgery	MC1273; Radiation therapy and docetaxel in treating patients with HPV-related oropharyngeal cancer (NCT01932697) [48]	Phase II	TORS followed by 30 (ENE^−^) or 36 Gy (ENE^+^) with concurrent docetaxel
	MC1675: DART-HPV; Evaluation of de-escalated adjuvant radiation therapy for human papillomavirus (HPV)-associated oropharynx cancer (NCT02908477) [49]	Randomized phase III	TORS followed by 30–36 Gy with docetaxel versus 60 Gy +/− cisplatin
	ECOG 3311; Transoral surgery followed by low-dose or standard-dose radiation therapy with or without chemotherapy in treating patients with HPV positive stage III-IVA oropharyngeal cancer (NCT01898494) [43, 44]	Randomized phase II	TORS followed by risk-stratified treatment; intermediate risk patients randomized to 50 Gy versus 60 Gy

**Abbreviations:** TORS, transoral robotic surgery; RT/CRT, radiotherapy/conformal radiotherapy; ENE, extranodal extension.

## Current Literature Supporting Proton Therapy for Head and Neck Cancer

Head and neck cancers are a diverse group of tumors that are usually in near proximity to critical structures. Although conventional photon (x-ray) radiotherapy (RT) can achieve favorable oncologic outcomes, it also unavoidably delivers higher radiation doses to the adjacent healthy organs, which can lead to serious toxicities and affect the quality of life (QoL) of these patients. On the other hand, particle beam therapy offers dosimetric advantages over photon RT because of its sharp lateral penumbra and steep distal falloff, minimizing integral radiation dose to healthy tissues and allowing treatment deintensification while improving toxicity profiles. The advantages in clinical practice of particle beam therapy for the treatment of head and neck cancers have been pointed out in multiple reports over the past several years. Frank et al [7] reported their experience using multifield-optimized intensity-modulated proton therapy (IMPT) in the management of head and neck malignancies. After a median follow-up of 28 months, all patients completed treatment with no interruptions or hospitalizations and with minimal associated toxicity (xerostomia and mucositis), despite most been treated with concurrent chemotherapy and comprehensive radiation fields (skull base to the clavicles), for an overall clinical complete response rate of 93.3% [7].

A case-matched analysis from MD Anderson Cancer Center compared the use of IMPT (n = 50) versus intensity-modulated radiation therapy (IMRT) (n = 100) for patients with oropharynx cancer [8]. With a median follow-up of 32 months, no statistical differences in overall survival (OS) or progression-free survival (PFS) were observed, although rates of feeding tube dependency and severe weight loss at 3 months and 1 year were significantly reduced when IMPT was employed [8].

Moreover, Sharma et al [9] evaluated patient-reported QoL outcomes of patients with oropharyngeal squamous cell carcinoma treated with transoral robotic surgery (TORS), followed by adjuvant pencil beam scanning proton therapy (31 of 64; 48%) or volumetric-modulated arc therapy (VMAT) (33 of 64; 52%). Patient-reported QoL (European Organisation for Research and Treatment of Cancer [EORTC] QLQ-c30 version 3, EORTC OLO-H&N35, and the Groningen (GRIX) Xerostomia, Work Status, and Performance Status Scale–Head and Neck Cancer questionnaires) reports were prospectively collected at the time of initial RT consultation (pretreatment), and at 3-, 6-, and 12-month follow-ups. The general health domain, physical and role function, overall xerostomia, dental issues, head and neck pain, and fatigue scores were determined by the EORTC questionnaire, whereas the GRIX assessment helped to analyze and separate, different components of xerostomia, such as grades of sticky saliva and variances between day and night mouth dryness. For purposes of their analysis, composite scores examining specific domains were linearly converted to a 0 to 100 scale and considered clinically significant with a score difference of 10 in the composite scale. As expected, proton therapy resulted in significant sparing and/or mean radiation dose reduction of oral cavity structures (upper and lower lips, tongue, hard palate, and buccal mucosa), and salivary glands (contralateral parotid and sublingual glands). To determine whether these dosimetric advantages correlated with patient-reported outcomes (PROs), the EORTC and GRIX questionnaires were applied. Reassuringly, there was a clear relationship between PROs and the observed dose reduction to healthy organs at risk with the use of proton therapy, translating into significantly less xerostomia, dental problems, head and neck pain, physical and role function at multiple time points (3, 6 and 12 months), reflecting objectively in QoL metrics, the clinical advantages of using of PBT in the management of these tumors [9].

Furthermore, reducing incidental radiation dose to neighboring critical structures with the use of proton therapy can translate into additional clinical benefits. Sio et al [10] performed a comparative analysis of PROs from the use of IMPT versus IMRT with concurrent chemotherapy for the treatment of oropharyngeal cancers. An MD Anderson Symptom Inventory Head and Neck Cancer module was completed at baseline, during treatment (acute), within 3 months after treatment (subacute), and later (chronic phase). The top 5 symptoms were food-taste problems (mean score, 4.91 [0–10 scale]), dry mouth (4.49), swallowing/chewing difficulties (4.26), lack of appetite (4.08), and fatigue (4.00). After analysis, changes in taste and appetite during the subacute and chronic phases were less with the use of IMPT. Additionally, they also found lower mean scores in the top-5 reported symptoms, 5.15 ± 2.66 for IMPT compared with 6.58 ± 1.98 for IMRT (p = 0.013) during the subacute phase [10], which is a critical period in the recovery process for patients with head and neck cancer, and where lingering side effects would typically translate into increased chronic toxicity or morbidity, further hospitalizations, monetary costs, and a significant delay in the healing process of these patients. The clinical benefit of proton therapy for oropharyngeal cancer is currently being investigated in a prospective randomized trial, which should shed more light on this question (NCT01893307, “Randomized trial of intensity-modulated proton beam therapy (IMPT) versus intensity-modulated photon therapy (IMRT) for the treatment of oropharyngeal cancer of the head and neck” [11]).

It is well known that the definitive management of locally advanced head and neck cancers often involves combined modality therapy, either with chemoradiotherapy (CRT) or surgery followed by adjuvant therapies upon high-risk pathologic features. Recently, the importance of treatment-induced lymphopenia and its association with higher rates of distant metastasis and decreased overall survival has been recognized, translating to worse clinical outcomes. Lymphocytes are highly radiosensitive, with a 50% lethal dose in the range of 1 to 2 Gy [12], and with circulating lymphocytes predicted to receive mean doses around 2 Gy during a conventional course of fractionated photon RT [13]. Interestingly, RT may have a stronger effect on the induction of lymphopenia when compared with chemotherapy [14]. Routman et al [15] evaluated whether RT modality and technique, PBT versus photon beam therapy, could affect the severity of treatment-induced lymphopenia. What they found were higher rates of G4 lymphopenia (absolute lymphocyte count < 200/mm^3^) in patients treated with curative-intent photon RT and concurrent chemotherapy, in comparison with their peers treated with proton RT with chemotherapy (56% versus 22%, p < 0.01). These results appear to be related to a meaningful integral dose reduction with the use of proton therapy [15]. Moreover, these findings can potentially translate into practice in the way of better tolerance to treatments, lower risk of infections and hospitalizations, and improved oncologic and functional outcomes. To date, there is no clear understanding of the precise nature of the relationship between antitumor immunity effect and specific RT modality or technique.

A topic of constant debate among oncologists, institutions, and insurance companies is whether the advantages of particle beam therapy could translate in a tangible cost-benefit. A study from the Netherlands [16] used a decision-analytic Markov cohort model to examine and comparative costs, quality-adjusted life years (QALYs), and disease- and toxicity-free life years (DTFLYs) of 3 treatment strategies: IMPT for all patients, IMRT for all patients, and a mixed IMPT-IMRT group in which patients for whom IMPT was expected to be cost effective received that treatment modality, whereas the remaining received IMRT. The evaluated population consisted of patients with locally advanced cancer and oral cavity, laryngeal, and pharyngeal primaries. The estimated occurrence of xerostomia and dysphagia was less in the IMPT for all patients group at 1 year. Moreover, IMPT for all patients was also the most effective (6.620 QALYs, 5.800 DTFLYs) and most expensive 50,989 (US $60,167) modality. The IMRT for all patients was the least effective (6.520 QALYs, 4.197 DTFLYs) and least expensive 41,038 (US $48,424). For the mixed IMPT-IMRT group restricting IMPT to patients for whom IMPT was expected to be cost effective yielded 6.563 QALYs, and 4.875 DTFLYs at an estimated cost of 43,650 (US $51,507). Their results suggest that the use of IMPT in properly selected patients with head and neck cancer can be an effective modality with nearly equivalent costs as IMRT [16].

Verma et al [17] performed a cost-effectiveness systematic review of the use of PBT after evaluating 18 original investigations of different disease sites, including head and neck cancers. A total of 3 studies were included in the analysis of head and neck malignancies, including the one from Raemakers et al [16] previously discussed in detail in this review, suggesting that IMPT could decrease specific treatment-related toxicities, such as xerostomia and dysphagia, using the Radiation Therapy Oncology Group grading system for healthy tissue complications. Similarly, a Swedish study [18] used a Markov cohort-simulation model to estimate the life of patients treated with radiation, with cost and QALYs as the primary outcome measures. That cost-effectiveness model was associated with a relatively large gain of 1.02 QALYs, and only a small increase in cost at 3,887 (US $4,703) for proton therapy in comparison to x-ray therapy. Interestingly and worth mentioning, the study lacked toxicity and QoL data at the time of analysis, making the clinical applicability of the results questionable. Additionally, the systematic review included a literature-based, nonmodeling cost-calculation study for head and neck cancers [19], estimating increased treatment costs with the use of proton therapy compared with IMRT; however, in parallel to the Swedish study [18], no cost-effectiveness analysis was made for assessment of outcomes or toxicities, in addition to a lack of specification of the tumors treated, limiting the conclusions of their analysis. Verma et al [17] concluded that PBT could offer superior cost effectiveness in patients with head and neck cancer, particularly for properly selected patients with high risk of acute mucosal toxicities.

The effect on patient life of the treatment-related side effects caused by incidental radiation doses to healthy organs is broad and its often-underestimated, multiple physical and mental aspects, can be reflected even many years after treatment in the form of chronic complications, disabilities, additional medical procedures or hospitalizations, increasing costs substantially. In the past several years, there is a growing amount of data about the advantages and clinical benefits of PBT, with an increase in the availability of highly specialized centers and decreased construction costs, we can envision the accessibility to these promising therapies continuing to increase.

## Volume Deintensification

Historically, oropharyngeal carcinomas have been treated with either open surgery (lip-split mandibulotomy) or primary RT. Lately, newer surgical techniques, such as TORS and transoral laser microsurgery, have emerged as alternative treatment options with comparable oncologic outcomes and less morbidity. In the era of HPV-OPSCC, deintensification of therapy is an area of major interest. Currently, a common and often the only indication of the need of adjuvant therapies after transoral surgery for HPV-OPSCC is the presence of cervical lymphadenopathy. Therefore, we can entertain the idea of omitting the primary site, if and only if, there is an absence of high-risk pathologic features at the surgical bed.

The Mayo Clinic reported their initial experience of 40 patients with favorable-risk oropharyngeal carcinoma treated with transoral surgery, followed by adjuvant IMRT to only the neck with omission of the primary site [20]. Patients were considered favorable risk if they had negative margins, no lymphovascular invasion (LVI) or perineural invasion (PNI) at the primary site, with indications of adjuvant RT because of multiple positive lymph nodes, lymph nodes > 3 cm, and/or the presence of extranodal extension. After a median follow-up of 51 months, no local failures were documented, with only one regional failure, for a 97.5% locoregional control and 97% OS rate at 4 years. Acute G3 toxicity was reported in only 1 patient (dysphagia), with no G4 or G5 toxicities documented. The mean incidental doses to the primary tonsillar and base of the tongue surgical beds were 4320 centigray (cGy) (SD ± 480 cGy), and 4060 cGy (SD ± 420 cGy), respectively. This preliminary data suggest this treatment approach is feasible and safe in this selected favorable-risk group of patients, opening the question of whether other RT modalities, such as proton therapy, can even further deintensify therapy [20].

After these encouraging preliminary results, Mayo Clinic opened an observational prospective clinical trial of mucosal sparing PBT in resected oropharyngeal tumors (NCT02736786 [21]). Eligible patients were those who underwent transoral surgical resection using frozen-section margin analysis [22], with negative margin resection and no other adverse features (PNI and LVI), with pathologic stage T1 and T2, N1 to N3, M0 disease, and indications of adjuvant RT based on positive neck lymphadenopathy. The trial [21] has completed accrual, enrolling 61 patients. Currently, primary investigators are awaiting longer follow-up to report their results.

Recently, the University of Pennsylvania group reported the safety and efficacy results of a phase II trial omitting the resected primary tumor bed from the radiation treatment field after TORS for HPV-related squamous cell carcinoma of the oropharynx (the AVOID trial, NCT02159703 [23]) [24]. Sixty patients with T1 and T2 tumors and favorable pathologic features at the primary site (negative surgical margins ≥ 2 mm, no perineural invasion, and no lymphovascular invasion) and indications of adjuvant RT mainly because of lymph node involvement were included in this single-institution analysis. Postoperative delivered RT dose to the involved neck ranged from 60 to 66 Gy and 54 Gy for the uninvolved neck. Thirty-two patients (53%) were treated with IMRT technique, whereas 27 (45%) received proton therapy. All patients had unilateral selective neck dissection and received bilateral selective neck irradiation. Thirteen patients (22%) received adjuvant concurrent chemotherapy. After a median follow-up of 2.4 years, only 1 patient had recurrence at the primary site, for a 2-yr local control of 98.3%. An additional patient developed a regional neck recurrence (1.7%), and 2 other patients developed distant metastatic disease (3.3%). The 2-yr OS was 100%. Low rates of percutaneous endoscopic gastrostomy (PEG) tube dependence and late soft-tissue necrosis (3.3%) at the primary surgical bed were reported [24].

The concept of contralateral neck sparing with the use of photon therapy has been advocated in the past couple of decades for the management of well-lateralized, early stage oropharyngeal carcinoma, resulting in favorable oncologic outcomes, lower toxicity rates, and improved long-term function in well-selected patients [25]. Furthermore, the group from Washington University performed a prospective phase II trial eliminating postoperative radiation to the pathologically node-negative neck in patients with head and neck squamous cell carcinoma [26]. Patients with well-lateralized, early stage (T1–T2, N0–N2b) tonsil cancers with clinically negative contralateral necks were treated with ipsilateral postoperative RT and were excluded from the study [26]. A total of 72 patients were enrolled after undergoing resection of the primary tumor and bilateral lymph node neck dissections, both in a combined or staged manner, and after confirmation of a PN0 neck, with indication of postoperative RT based on pathologic high-risk features (close or positive surgical margins, PNI, LVI, ≥ 1 positive lymph nodes, extranodal extension). Thirty-seven patients (51%) had an oropharyngeal primary tumor. p16 status was tested in 35 (49%) oropharyngeal tumors; of which, thirty-two (91%) were positive. The high-risk primary tumor and involved nodal bed received 60 to 66 Gy, and the elective involved neck nodal basins received 52 to 54 Gy in 33 fractions. With a median follow-up of 53 months, only 2 patients were documented with treatment failure on the PN0 unirradiated neck, with both also experiencing local treatment failures, for a regional control of 97% [26].

With more-accurate diagnostic imaging modalities and improvements in radiation-delivery techniques, such as IMPT, the idea of further deintensification therapy for these patients has become more tangible. In the past few years, several institutions have reported their experiences with the management of locally advanced head and neck tumors and the use of PBT [27–29]. The University of Washington [27] reviewed 46 patients that received either definitive IMPT (n = 28, 61%) or postoperative IMPT (n = 18, 39%); of whom, 16 patients (35%) received unilateral neck RT for the management of HPV-related, locally advanced oropharyngeal squamous cell carcinoma. At a median follow-up of 19.2 months, the OS and PFS rates were 95.7% and 93.5%, respectively, with no local, regional, or marginal recurrences. As expected because of the inherent physical properties of charged particles, patient candidates for unilateral neck RT had significantly lower radiation mean doses to the contralateral and midline critical structures, translating to reduced treatment toxicities [27]. Similarly, Memorial Sloan Kettering Cancer Center [29] reported their comparative results with PBT (18 of 41; 44%) versus IMRT (23 of 41; 56%) for the management of major salivary gland cancers or cutaneous squamous cell carcinoma in patients who underwent ipsilateral irradiation. Dosimetrically, PBT plans achieved lower doses to organs at risk when compared with IMRT, correlating with lower rates of acute grade ≥ 2 dysgeusia, mucositis, and nausea [29].

Manzar et al [30] reported a comparative analysis of acute toxicities and PROs between IMPT and VMAT for the treatment of oropharyngeal cancer. All patients underwent prospective data collection using PROs, the EORTC-QLQ-H&N35 questionnaire, and provider-assessed toxicities (Common Terminology Criteria for Adverse Events, version 4.03). PEG-tube placement, hospitalization, and narcotic-use data were retrospectively collected; 305 patients were included in the analysis (IMPT, n = 46, 15%; VMAT, n = 259, 85%), for whom a total of 44 patients (14%) received unilateral neck irradiation (IMPT, n = 6, 14%; VMAT, n = 38, 86%). Pretreatment and end-of-treatment scores were compared. In the entire cohort, mean radiation doses to organs at risk (larynx, pharyngeal constrictors, parotid gland, and oral cavity) were significantly reduced with the use of IMPT, translating, in clinical practice, to lower rates of mucositis, xerostomia, dysgeusia, dysphagia, PEG-tube placement, pain or the use of narcotics, and hospitalization days, with improvement on PROs, particularly for those treated with definitive-intent IMPT [30].

Another mechanism to further deintensify therapy and improve the therapeutic ratio is sparing different lymph node levels from RT. The University of Chicago developed a phase II trial of dose and volume de-escalation for the treatment of HPV-OPSCC (OPTIMA trial; NCT02258659 [31]) [32]. Sixty-two patients were stratified as low-risk (n = 28; 45%) (T1–T3, N0–N2b unless bulky N2b conglomerate [≥ 6 cm], and ≤ 10 pack-y history), or high-risk (n = 34; 55%) (T4, N2c–N3, bulky N2b disease, or > 10 pack-y history). Before treatment, patients underwent panendoscopy and positron emission tomography/computed tomography. All patients received 3 cycles of induction chemotherapy with carboplatin and nanoparticle albumin-bound paclitaxel (nab-paclitaxel). Low-risk patients with ≥ 50% response evaluation criteria in solid tumors were followed by RT alone (VMAT or IMRT), 50 Gy in 25 fractions over 5 consecutive weeks (RT50). Low-risk patients with < 50% but ≥ 30% response and high-risk patients with ≥ 50% response received CRT, 45 Gy at 1.5 Gy twice-daily with concurrent chemotherapy TFHX (paclitaxel, 5-fluorouracil, hydroxyurea, and twice-daily RT administered every other week) (CRT45). Low-risk patients with < 30% response, high-risk patients with < 50% response, or patients with documented disease progression were managed with CRT for a total dose of 75 Gy at 1.5 Gy twice-daily RT and TFHX (CRT75). Radiation target volumes included preinduction chemotherapy of gross disease plus 1.5-cm additional expansion, which was limited to the first echelon of uninvolved nodes. After a median follow-up of 29 months, the 2-year OS and PFS were 100% and 95% for low-risk patients and 97% and 94% for high-risk patients, respectively. Regarding treatment-related toxicities, grade ≥ 3 mucositis occurred in 30% of the RT50 group, 63% in the CRT45, and 91% in the CRT75 groups. Similarly, rates of need for PEG-tube placement were 0%, 31%, and 82% for RT50, CRT45, and CRT75 groups, respectively [32].

## Radiation Dose Deintensification for HPV-OPSCC

The physical properties of a proton beam, combined with volume reduction strategies, allow for substantial reductions in healthy-tissue radiation exposure. Nevertheless, patterns of failure data for oropharynx cancer often require treatment volumes that unavoidably cover at least a portion of healthy structures. For example, coverage of retropharyngeal lymph nodes invariably involves at least the lateral pharyngeal constrictor muscles. Attempts to avoid these at-risk areas, such as the medial parotid gland, for example, can be linked with increased risks of regional nodal recurrence. These overlapping areas between at-risk volumes and healthy tissue structures comprise unavoidable areas of toxicity with radiation therapy, particularly in serial structures. It is in these settings that proton therapy synergizes with radiation dose-reduction efforts underway with HPV-OPSCC. This section will review how the clinical benefit from radiation dose-reduction strategies can be maximized with proton therapy.

The most straight-forward strategy for radiation dose deintensification is to have the radiation dose reduction as the sole alteration in the treatment strategy. For example, a phase II study by Chera et al [33] investigated a course of standardly fractionated IMRT with weekly cisplatin to a dose of 60 Gy, rather than 70 Gy. That radiation schedule yielded a 2-year locoregional control and OS rate of 95%, comparable to historic controls. In comparison, NRG-HN002 was a randomized phase II trial (NCT02254278 [34]) comparing standardly fractionated 60 Gy with concurrent cisplatin vs 60 Gy of accelerated radiation therapy alone over 5 weeks [35]. At 2.6 years of median follow-up, the projected 2-year PFS was 90.5% for concurrent cisplatin and 87.6% for radiation alone [35]. Notably, even with a relatively modest 14% dose reduction, both trials detailed significant benefits in grade ≥ 3 adverse events and 1-year MD Anderson dysphagia inventory scores when compared with historic comparators [36].

Given the larger volume of disease, radiation doses for definitive treatment are necessarily greater than radiation doses required for subclinical or microscopic disease. Because long-term healthy-tissue toxicity is often linked with cumulative radiation dose, deintensification strategies using multimodality therapy to debulk disease burden have also been investigated. Induction chemotherapy provides one means for simultaneously debulking a tumor when chemoselecting patients for radiation deescalation. For example, a phase II trial by Chen et al [37] followed induction chemotherapy with 54 Gy and weekly paclitaxel to responders and 60 Gy for nonresponders. Similarly, the Eastern Cooperative Oncology Group (ECOG) 1308 trial (NCT01084083 [38]) used induction chemotherapy, followed by 54 Gy and cetuximab for responders and 69.3 Gy for nonresponders [39]. Although induction chemoselection strategies generally have lower radiation doses when compared with definitive RT trials, the incremental benefit in dose reduction must be balanced with the toxicity of additional therapy, particularly hematologic toxicities. Furthermore, 45% of patients in Chen et al [37] and 30% of patients on ECOG 1308 [39] did not have sufficient tumor shrinkage to qualify for the largest radiation dose reduction. Those patients thus received a potential treatment intensification from the additional chemotherapy doses. Nevertheless, even without radiation-dose deintensification, induction chemotherapy may allow for treatment volume reductions that are particularly germane with proton therapy. Although current clinical guidelines based upon expert consensus still recommend treating the preinduction volume in the high-dose volume, emerging data for oropharyngeal cancer suggests that restricting the high-dose volume to the postinduction volume does not compromise locoregional control [40, 41]. Minimally invasive transoral surgery provides another pathway for larger radiation-dose deintensification. Similar to the use of induction chemotherapy, careful patient selection for appropriate surgical cases must be used because surgical defects from wide-margin resections could be a larger driver of QoL issues compared with radiation dose [42]. However, in appropriately selected patients, particularly with exophytic, early stage tumors, surgical resection allows for larger radiation deintensification because the residual disease burden is expected to be either microscopic or subclinical volumes of disease. For example, ECOG 3311 (NCT01898494 [43]) randomized patients with intermediate risk factors after surgery to 60 versus 50 Gy of adjuvant radiation while maintaining the standard 60 to 66 Gy of adjuvant chemoradiation for patients with high-risk disease [44]. The deintensified intermediate-risk cohort maintained a 2-year PFS rate of 95%, whereas the high-risk cohort receiving standard adjuvant treatment had a 2-year PFS rate of 90.5% [44]. For more aggressive deintensification efforts, the Mayo Clinic investigated deescalating margin-negative, postoperative patients to 30 to 36 Gy with a 2-year locoregional control and PFS rate of 96.2% and 91.1%, respectively [45]. This deintensification regimen is currently awaiting results from a completed phase III randomized trial. These trials demonstrate how surgical resection can potentially allow for degrees of radiation deintensification that would be difficult to achieve with other modalities and synergize well with proton therapy.

## Conclusion

Proton therapy offers a number of deintensification strategies both off and on a clinical trial. The inherent dose characteristics of PBT translate into healthy-tissue sparing for oropharyngeal cancer treatment, even with standard treatment volumes and doses. Furthermore, the reduced low-dose spillover may translate into hematologic and immunologic benefits, which should be explored more prospectively. These dose characteristics may result in cost savings because of reduced side effects and better toxicity management in the long term.

Off of a clinical protocol, proton therapy can leverage prospective data concerning volume reduction. In the induction setting, generating the high-dose target volume based on the postinduction primary and nodal disease appears safe and reasonable. Similarly, postsurgical volume modifications can be optimized using proton therapy. Avoidance of the primary site for early stage tumors and avoidance of the dissected, N0 contralateral neck have both shown utility in prospective studies. Although deintensification efforts for HPV-related oropharynx cancer continue to mature, these efforts are also still investigational. At this time, we recommend enrolling patients in clinical trials similar to those listed in the Table, which also allows for proton therapy. These trials will help provide increased clarity about the role of proton therapy in treatment deintensification. Clinical trials looking at radiation dose deintensification in the definitive, induction, and adjuvant settings are all underway. These studies complement the role of proton therapy because clinical target volumes often unavoidably overlap with healthy-tissue structures. Clinical trials leveraging proton therapy with both volume and dose deintensification should be further explored as part of individualized medicine protocols.
